# The Kuopio idiopathic normal pressure hydrocephalus protocol: initial outcome of 175 patients

**DOI:** 10.1186/s12987-019-0142-9

**Published:** 2019-07-25

**Authors:** A. Junkkari, A. J. Luikku, N. Danner, H. K. Jyrkkänen, T. Rauramaa, V. E. Korhonen, A. M. Koivisto, O. Nerg, M. Kojoukhova, T. J. Huttunen, J. E. Jääskeläinen, V. Leinonen

**Affiliations:** 1 Neurosurgery of NeuroCenter, Kuopio University Hospital (KUH) and University of Eastern Finland (UEF), POB 100, 70029 Kuopio, Finland; 2 Department of Pathology, KUH and UEF, Kuopio, Finland; 3 Neurology of NeuroCenter, KUH and UEF, Kuopio, Finland; 4 Unit of Clinical Neuroscience, Neurosurgery, University of Oulu, Oulu, Finland; 5 MRC Oulu, Oulu University Hospital, Oulu, Finland

**Keywords:** Normal pressure hydrocephalus, Outcome, Tap test, Infusion test, Comorbidity

## Abstract

**Background:**

The Kuopio University Hospital (KUH) idiopathic normal pressure hydrocephalus (iNPH) cerebrospinal fluid (CSF) shunting protocol is described together with the initial outcomes of 175 patients with probable iNPH treated according to this protocol from a defined population. Our secondary aim was to display the variety of differential diagnoses referred to the KUH iNPH outpatient clinic from 2010 until 2017.

**Methods:**

Patients were divided into four groups according to the prognostic tests: tap test (positive or negative) and infusion test (positive or negative). The short-term outcome was compared between groups. The 3-month outcome following shunt surgery was assessed by measuring gait speed improvement, using a 12-point iNPH grading scale (iNPHGS) and the 15D instrument.

**Results:**

From 341 patients suspected of iNPH, 88 patients were excluded from further research mostly due to deviation from the protocol’s gait assessment guidelines. Hence 253 patients with suspected iNPH were included in the study, 177/253 (70%) of whom were treated with a CSF shunt. A favorable clinical outcome following surgery was observed in 79–93% of patients depending on the prognostic group. A moderate association (Cramer’s V = 0.32) was found between the gait speed improvement rate and the prognostic group (X^2^, p = 0.003). Patients with a positive tap test had the highest gait speed improvement rate (75%). In addition, an improvement in walking speed was observed in 4/11 patients who had both a negative tap test and a negative infusion test. Other outcome measures did not differ between the prognostic groups. Conditions other than iNPH were found in 25% of the patients referred to iNPH outpatient clinic, with the most prevalent being Alzheimer’s disease.

**Conclusions:**

Our results emphasize the importance of a systematic diagnostic and prognostic workup especially in cases with an atypical presentation of iNPH. Additional diagnostic testing may be required, but should not delay adequate care. Active surgical treatment is recommended in patients with a high clinical probability of iNPH. Other neurological conditions contributed to most of the non iNPH diagnoses.

**Electronic supplementary material:**

The online version of this article (10.1186/s12987-019-0142-9) contains supplementary material, which is available to authorized users.

## Introduction

Idiopathic normal pressure hydrocephalus (iNPH) is a progressive neurological disorder, affecting the aged population, which can be ameliorated by cerebrospinal fluid (CSF) shunting [1, 2]. A suspicion of iNPH rises, when patients exhibit a progressive worsening of gait, cognitive impairment and urinary incontinence, accompanied with ventricular enlargement (ventriculomegaly) demonstrated by computed tomography (CT) or magnetic resonance imaging (MRI) of the brain [1, 2]. It has been recently hypothesized that ventriculomegaly might be a sign of early neurodegeneration [3]. Since several conditions may feature a similar gait disorder [3, 4] and ventriculomegaly [3, 5] thorough diagnostic evaluations should be performed in collaboration with neurologists, neuroradiologists and neurosurgeons [3–6]. Due to the progressive nature of iNPH, patients should be treated without unnecessary delays after establishing the diagnosis, since the condition worsens over time [7, 8]. However, also the response rate for CSF shunting seems to begin to decrease on average after 6 months following surgery [9], possibly indicating the progression of iNPH or its comorbidities [10]. It has also been suggested that a non-sustained response may indicate another condition than iNPH [3].

**Fig. 1:**
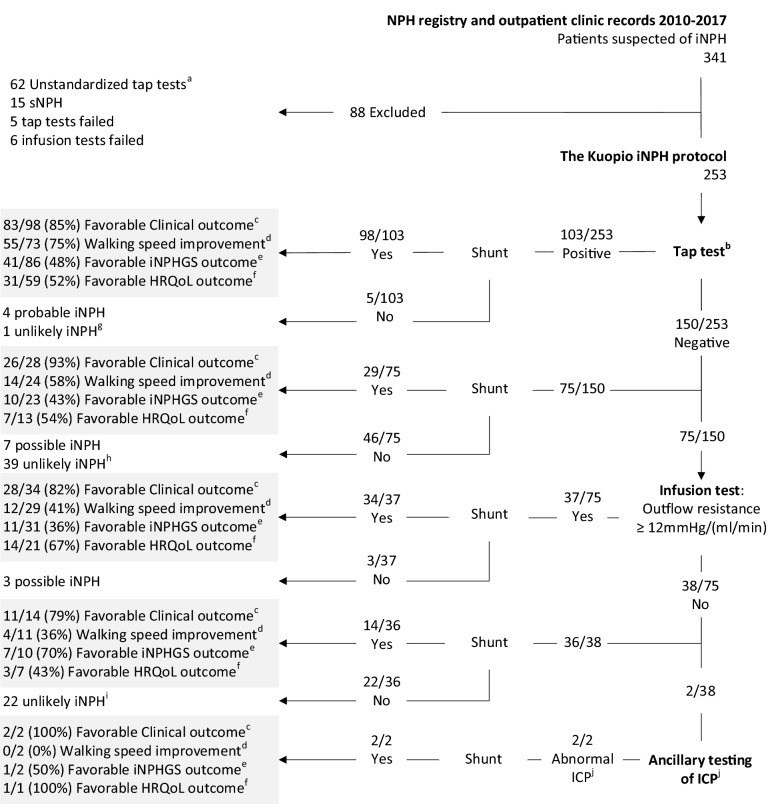
Flowchart of the study population. The initial outcome 3 months postoperatively has been highlighted in grey in each prognostic group. The number of observations, ratios and percentages have been given to account for any missing data. ^a^Tap test evaluation based only on clinician’s judgement. ^b^Description of the performance and interpretation of tap test are found in (Table 1). ^c^Improvement of any core symptoms (gait, cognition and urinary incontinence) assessed by neurosurgeon. ^d^At least 20% improvement in average gait speed, gait test task and evaluation described in (Table 1). ^e^Reduction in the iNPHGS total score at least by a single point. ^f^At least 0.015 improvement in 15D score. ^g^1 Ventriculomegaly (congenital or unclear etiology). ^h^8 VAD or CVD,7 AD, 7 AD + VAD, 3 Cognitive impairment or dementia of unspecified etiology, 3 Ventriculomegaly (congenital or unclear etiology), 3 PD (2 vascular, 1 idiopathic), 2 Drug induced parkinsonism or psychiatric condition, 2 traumatic brain injury, 1 FTD, 1 Spinal stenosis, 1 arthrosis, 1 LBD. ^i^7 AD, 5 spinal stenosis, 3 Cognitive impairment or dementia of unspecified etiology, 3 VAD or CVD, 2 Drug induced parkinsonism or psychiatric condition, 1 LBD, 1 Vertigo. ^j^Patients underwent 24-h intraventricular measurement of ICP, interpretation of the monitoring is described in (Table 1). *iNPH* Idiopathic normal pressure hydrocephalus, *sNPH* secondary normal pressure hydrocephalus, *ICP* intracranial pressure, *VAD* vascular dementia, *CVD* cerebrovascular disease, *AD* Alzheimer’s disease, *PD* Parkinson’s disease, *iPD* idiopathic PD, *LBD* Lewy’s bodies disease

In three decades the methods and criteria for diagnosing iNPH and predicting the outcome of CSF shunting, while not perfect, have become more robust and less invasive [1, 2]. During this time, a gradual adaptation to the iNPH guidelines and literature has modified the practice in Kuopio University Hospital’s (KUH) NPH outpatient clinic: From 1991 until 2010 the KUH protocol included a 24-h intraventricular pressure monitoring from all patients with suspected iNPH. In early 2010, after the adaptation of tap-test, infusion testing and motivation to decrease risks involved with direct intracranial pressure (ICP) monitoring, a three-step prognostic test protocol was launched (Fig. 1, Table 1), the KUH iNPH protocol. Our aim is to describe KUH iNPH protocol and the initial outcomes of 175 patients with probable iNPH who were treated based on this protocol.

**Table 1:** Use and Interpretation of prognostic or diagnostic tests in KUH iNPH protocol

Prognostic or diagnostic test	Performance	Interpretation	Action
Tap test	Gait task: Walking 10 meters, rotating and returning to the starting point (20 m in total). Repeated twice prior and twice after the CSF removal by lumbar puncture CSF drainage: 20–40 ml of CSF is removed by lumbar puncture in a sitting position. Patient rests 1 h in supine position before the repetition of the gait task Evaluation: Examining the gait in a standardized manner. The average gait speed (meters/second), the number and the length of steps prior to and after CSF removal are calculated	Positive^a^ if there is at least 20% improvement in average gait speed. Negative if less	If positive, patient is referred for CSF shunt treatment, if the patient is willing, and if there are no contraindications Negative test does not exclude favorable CSF shunt treatment outcome. Such patients are referred to the infusion test if willing
Infusion test	CSF outflow resistance via lumbar catheter by measuring changes in ICP caused by continuous or pulsatile infusion of Ringer solution	Positive^b^ if: Outflow resistance is ≥ 12 mmHg/(ml/min). Negative if not	If positive, patient is likely to suffer from iNPH, and thus may benefit from CSF shunt treatment. If test is negative, patient is unlikely to have iNPH, but some iNPH patients may have normal findings
Continuous measurement of ICP	24 h, intraventricular measurement of ICP	Positive if: (a) a basal intracranial pressure is above 10 mmHg or (b) the presence of any A waves or (c) more than 30% B waves during the monitoring	If positive, patient is likely to suffer from iNPH, and thus may benefit from CSF shunt treatment. If test is negative, patient is unlikely to have iNPH, but some iNPH patients may have normal findings

*CSF* Cerebrospinal fluid, *iNPH* idiopathic normal pressure hydrocephalus, *KUH* Kuopio University Hospital, *ICP* intracranial pressure, *ml* milliliter, *min* minute, *mmHg* millimeter of mercury

^a^There are no uniform guidelines how to measure and what is the minimal clinically significant gait improvement after CSF removal

^b^Stricter outflow resistance limits exist (13, 19, 20)

Our secondary objective was to examine whether the patients selected to the treatment on each step of the prognostic test protocol, differentiated from each other in terms of clinical appearance and severity of the illness, and to describe the initial outcomes in each prognostic group. Our tertiary aim was to display the variety of differential diagnoses referred to the KUH iNPH outpatient clinic from 2010 until 2017.

### Current prognostic and differential diagnostic tests for iNHP

The lumbar tap test (LTT) has been used to temporarily emulate the function of a CSF shunt and to predict the outcome of treatment [2, 11–13] (Table 1). It has been demonstrated that a larger volume of drained CSF during LTT does not provide additional value [14] but some researchers have suggested that a longer observation time (up to 24-h) may be used to increase the sensitivity of the LTT [15]. In addition to measuring walking speed in the LTT, a timed up and go (TUG) test can also be used [16, 17]. As an alternative to a single lumbar puncture, another option is to continuously drain CSF over several days removing a total volume of 300–500 ml of CSF, also known as external lumbar drainage (ELD) [2, 13]. Despite the various test methods, reports on the minimal clinically significant improvement in gait speed after LTT or ELD are scarce [13]. However, patients with a strong clinical suspicion of iNPH but negative results in LTT should not be denied CSF shunt surgery, but undergo further testing of CSF hydrodynamics [11–13, 18]. For example in iNPH, elevated CSF outflow resistance may be observed in a lumbar infusion test [13, 18–20]. Even though the diagnostic and prognostic workup of iNPH can be enhanced with the infusion test, there still remain patients with iNPH who potentially could benefit from CSF shunting despite a normal CSF outflow resistance in the infusion test [12, 13, 18, 20]. As a further test, continuous direct monitoring of ICP has been used, but in addition to the invasive nature of the procedure, the additional prognostic value of the different abnormalities observed during monitoring, such as B waves or pulsatile ICP, have not been confirmed [2, 13, 21]. Also more sophisticated computerized methods merging multimodal data, such as Disease State Index (DSI), have problems in predicting outcome of CSF shunting in patients with iNPH [22].

## Methods

### Patients

The permission for the research was received from the Research Ethics Board of KUH. The study was conducted according to the Declaration of Helsinki and all patients provided informed consent. People suspected to suffer from iNPH were primarily examined by a neurologist and referred for further neurosurgical investigations if the patient exhibited one to three symptoms possibly related to NPH (impaired gait, cognition or urinary continence) together with enlarged brain ventricles (Evans’ index > 0.3) in CT or MRI (Fig. 1) and without other explicit cause of the symptoms.

In early 2010, a three-step prognostic test protocol was launched (Fig. 1, Table 1). The use and interpretation of different prognostic or diagnostic tests used in KUH are presented in Table 1. In the three-step-protocol, an LTT is performed to all patients with suspected iNPH, where at least 20% improvement in average gait speed in repeated 10-m tests is considered as a positive result (Table 1). In the second phase, those with a negative LTT may undergo lumbar infusion test, where pathological findings (outflow resistance ≥ 12 mmHg/(ml/min)) [19] were considered as a positive result. In the third step, participants with a negative finding in both of the above-mentioned tests could further undergo a 24-h monitoring of intraventricular pressure (Table 1). Patients with negative findings at any stage of the prognostic testing could still be considered as candidates for shunt surgery based on clinical re-evaluation. Due to the invasive nature of the procedure, the number of patients referred to direct ICP monitoring has decreased over time.

#### Lumbar tap test

The LTT is used to temporarily emulate the function of a CSF shunt in order to predict the outcome of treatment [2, 11–13] (Table 2). This was done by examining the gait in a standardized manner before and 1 h after the removal of 20–40 ml CSF by lumbar puncture [2, 11, 13]. The KUH procedure for performing the LTT is described in detail in Table 1.

**Table 2:** Comparison of clinical characteristics and outcomes in 175 probable iNPH patients using different prognostic tests

Variables	Tap positive, infusion test not performed n = 98			Tap negative, infusion test not performed n = 29			Tap negative, infusion test positive n = 34			Tap negative, infusion test negative n = 14			Test statistics (df)	Test of strength	*p* value
	Number of participants	%	Number of observations if any missing data	Number of participants	%	Number of observations if any missing data	Number of participants	%	Number of observations if any missing data	Number of participants	%	Number of observations if any missing data			
Outcome															
Favorable clinical outcome 3 months postoperatively (yes)	83	85		26	93	28	28	82		11	79		2.23 (3)		0.527^a^
Walking speed improvement 3 months postoperatively (yes)	55	75	73	14	58	24	12	41	29	4	36	11	10.72 (3)	0.32^c^	*0.003* ^b^
Favorable INPHGS outcome 3 months postoperatively (yes)	41	48	86	10	43	23	11	36	31	7	70	10	3.86 (3)		0.278^b^
Favorable HRQoL outcome 3 months postoperatively (yes)	31	52	59	7	54	13	14	67	21	3	43	7	1.75 (3)		0.626^a^
Characteristics															
Age (at referral to the neurosurgical department)	74.3^f^	6.9^g^		73.5^f^	6.1^g^		74.1^f^	7.3^g^		72.4^f^	9.2^g^		0.66		0.882^d^
Sex (female)	43	44		13	45		18	53		8	64		2.58 (3)		0.460^b^
INPH related symptoms															
Impairment of gait	98	100		28	97		32	94		13	93		7.02 (3)		0.071^a^
Urinary incontinence or urge	80	82		23	79		26	76		14	100		3.87 (3)		0.276^a^
Impaired cognition	79	81		27	93		28	82		13	93		4.04 (3)		0.257^b^
Full triad	65	66		21	72		21	62		12	86		3.01 (3)		0.390^b^
Onset of iNPH related symtoms													4.05 (3)		0.256^b^
Onset a year or less from the referral	35	36	95	7	24		15	46	33	7	50				
Onset more than a year from the referral	60	64	95	22	76		18	54	33	7	50				
First symptom of iNPH															
Impairment of gait or imbalance	54	55		17	59		9	27		7	50		9.41 (3)	0.23^c^	0.024^b^
Cognition impairment	21	21		6	21		11	32		6	43		4.03 (3)		0.258^a^
Vertigo	10	10		0	0		7	21		1	7		9.59 (3)	0.21^c^	0.022^a^
Urinary incontinence or urge	8	8		3	10		3	9		0	0		2.57 (3)		0.463^a^
Other	5	5		3	10		3	9		0	0		3.09 (3)		0.379^a^
Severity of INPH related symptoms preoperatively															
INPHGS total score (0–12)	6.1^f^	2.6^g^	94	5.9^f^	2.8^g^		5.6^f^	2.8^g^		6.7^f^	2.6^g^	13	1.80		0.614^d^
Cognition impairment (MMSE score, 0–30)	22.0^f^	4.9^g^		22.9^f^	4.2^g^		22.5^f^	4.5^g^		23.1^f^	3.1^g^	13	0.73		0.865^d^
Comorbidity															
Presence of Aβ or HPτ found in the frontal cortical biopsy	56	60	93	9	34	26	17	55	31	5	45	11	5.71 (3)		0.126^b^
Surgical complications	9^h^	9		5^i^	17		1^j^	3		1^k^	7				0.259^a^

*iNPH* Idiopathic normal pressure hydrocephalus, *iNPHGS* iNPH Grading Scale, *MMSE* Mini-Mental State Examination, *Aβ* amyloid-beta, *HPτ* hyperphosphorylated tau

Statistically significant difference (Bonferroni correction, p < 0.0025) is italic

^a^Maximum likelyhoodratio Chi square test

^b^Pearson Chi square test

^c^Cramer’s V

^d^Intependent-Samples Kruskal–Wallis Test

^f^mean value

^g^standard deviation

^h^2 acute subdural hematomas caused by post-operative falling, 2 chronic subdural hematomas, 1 ventricular catheter displacement, 2 shunt infections, 1 peritoneal catheter displacement, 1 intracerebral hemorrhage

^i^3 chronic subdural hematoma, 2 shunt infections

^j^1 peritoneal catheter displacement

^k^1 peritoneal catheter displacement. Strength statistics were reported only for the statistically significant group differences

#### Infusion test

The infusion test was performed by a neurosurgeon using the Likvor CELDA^®^ System (19). Increased outflow resistance (≥ 12 mmHg/(ml/min) [19] was considered to support the diagnosis of probable iNPH.

### Shunt surgery

A ventriculoperitoneal shunt system was used in all patients. The ventricular catheter was placed from either a parieto-occipital or a frontal puncture with the latter being the only applied technique in recent years. The peritoneal catheter was placed via midline- or para-umbilical mini-laparotomy. At the beginning of the study period valves with a fixed pressure setting were used and later the policy was changed to installing adjustable valves in all patients.

### Biopsy procedure and immunohistochemistry

At surgery, three cylindrical cortical brain biopsies of 2–5 mm in diameter and 3–7 mm in length, were acquired preceding the insertion of CSF shunt proximal catheter, using biopsy forceps (until 2010) or disposable Temno Evolution^R^ TT146 biopsy needle (Merit Medical Systems Inc., South Jordan, UT, USA) (since 2010). The insertion point for the catheter was approximately 3 cm from the midline and anterior to the coronal suture. From all samples, a neuropathologist graded the presence of the immunoreactivity for amyloid-beta (Aβ) and hyperphosphorylated tau (HPτ) using light microscopy [23]. Patients were then further divided by the presence of pathology of any kind, the Aβ or HPτ observed in the frontal cortical biopsy (Table 2).

### Evaluation of outcome (3 months postoperatively)

#### Clinical outcome

A clinically-verified shunt response was assessed by a neurosurgeon at the outpatient clinic. [24] The patient was classified to be responsive to the CSF shunt if any improvement in the core symptoms (gait, cognition and urinary incontinence) was detected [24].

#### Walking speed improvement

A positive outcome in walking speed is considered as an improvement of at least 20%. The detailed performance and evaluation of the gait task is described in Table 2.

#### iNPH Grading Scale

To assess the severity of the symptoms of iNPH, a modified Finnish version of the 12-point iNPH Grading Scale (iNPHGS) was used [25]. INPHGS is a clinician-rated scale to separately estimate the severity of each of the triad symptoms with a scoring based on interviews with the patients or their caregivers and observations by the physician [25]. Lower scores represent less severe symptoms [25]. It has been estimated that even a reduction in the iNPHGS by a single point results in a clinically observable improvement in the patient’s condition [26].

#### 15D instrument

To assess the self-rated Health-related Quality of Life (HRQoL) outcome, a generic utility measurement, 15D instrument was used [27]. The 15D instrument has been recently described in detail in patients with iNPH [10]. The minimal clinically significant improvement in HRQoL, measured by 15D, was considered to be 0.015 [28].

#### Cognitive impairment

Cognition was evaluated by using the Mini-Mental State Examination (MMSE). MMSE ranges from 0 to 30, with lower scores indicating a greater cognitive decline [29].

### Statistics

The data was analyzed using the Statistical Package for Social Sciences (SPSS 22 for Windows, Version 22.0. IBM Corp., Armonk, NY, USA). Due to the non-normal distribution, independent-Samples Kruskal–Wallis test was used in multiple comparisons to estimate group differences in continuous variables. For non-continuous variables Pearson Chi square test was used. If the cell expecteds were 5 or less in more than 20% of cells, the table was tested with a maximum likelihood ratio Chi square test [30]. Cramer’s V was used as a post-test to test the strength of the association between the nominal variables. All tests for significance were two-sided, with probabilities of < 0.05 accepted as statistically significant. Stricter rejection criterion for α was performed using Bonferroni-correction (p = 0.05 divided by the number of comparisons) to take account multiple comparisons.

## Results

From 341 patients suspected of iNPH, 88 patients were excluded from further research (Fig. 1). The majority of exclusions (62/88) were caused by deviation from the protocol’s gait assessment guidelines (Table 1). 253 patients with suspected iNPH were included in the study (Fig. 1), 177/253 (70%) of whom were treated with a CSF shunt (Table 2). Patients were divided into groups according to the prognostic tests used (Fig. 1, Table 2): patients who were shunted on the basis of a positive LTT (98/177, 55%), negative LTT (29/177, 16%), negative LTT combined with positive infusion test (34/177, 19%) and to patients who had negative results in both above mentioned tests (14/177, 8%). Only 2 patients (2/177, 1.1%) were referred for ancillary direct invasive ICP monitoring after a negative LTT and infusion test, and thus were not included to statistical analyses.

### Outcome of CSF shunting

The clinical response to CSF shunting was high (79–93%) in all patient groups. A moderate association (Cramer’s V = 0.32) was found between the walking speed improvement rate and the prognostic group (X^2^, p = 0.003): the walking speed improvement rate was lower if the patient had a negative LTT, and was lowest in patients with both negative LTT and infusion test (4/11, 36%) (Fig. 1, Table 2). The INPHGS exhibited varying rates of success in each of the four groups that were not significantly different between the prognostic groups. In total, 14 probable or possible iNPH patients were not shunted (Fig. 1). In four cases, severe comorbidities prevented general anesthesia and 10 were due to patient’s refusal.

### Differences in clinical variables

The clinical symptomatology of iNPH was rather similar in all four groups (Table 2): gait impairment was present in 93–100%, urinary incontinence or urge was present in 76–100%, and cognitive impairment in 81–93% of patients. Gait impairment was observed in all patients who had a positive LTT (100%), whereas urinary incontinence and cognitive impairment were most frequently present in patients with a negative LTT and negative infusion test (100% and 93%). Patients who were treated with a CSF shunt regardless of the negative LTT or infusion test tended to present more frequently the full symptom triad (Table 2). These differences were, however, not statistically significant. The patient groups were indifferent in terms of age, cognitive impairment and the severity of iNPH (Table 2). There was no statistically significant difference in the onset of iNPH-related symptoms between the prognostic groups (Table 2).

After Bonferroni-correction for multiple testing, there was no significant difference between the groups when it come to the first presentation of iNPH. Prior to the correction, there was a weak association (Cramer’s V = 0.23) between the prognostic group and gait impairment or imbalance as the initial symptom (X^2^, p = 0.024). Prior to the correction, patients who did not undergo an infusion test seemed to have gait impairment or imbalance as the first symptoms more frequently than those to whom the infusion test was performed. Similarly, prior to the Bonferroni-correction, there was a weak association (Cramer’s V = 0.21) between the prognostic group and vertigo as the initial symptom (X^2^, p = 0.022). This atypical presentation seemed more prevalent in patients that underwent infusion testing.

The presence of Aβ or HPτ observed in the frontal cortical biopsy varied from 34 to 60%, and no statistically significant differences between the prognostic groups were observed.

Surgical complication rates did not differ between prognostic groups (Table 2). We did not observe significant/permanent complications caused by diagnostic or prognostic tests. Although not systemically collected for this study, we have observed few patients experiencing headache after LTT that required blood patch treatment. Similarly, a small group of patients experienced radiculating pain to lower limb during and shortly after LTT or infusion test, but this pain did not persist and did not require intervention.

Twenty-five percent (62/253) of the study participants had unlikely iNPH, with Alzheimer’s disease (AD) as the most frequent diagnosis (14/62, 23%) (Table 3). From 62 patients with unlikely iNPH, 11 (11/62, 18%) had vascular dementia (VAD) or cerebrovascular disease, seven (7/62, 11%) had VAD in addition to AD, six (6/62, 10%) had spinal stenosis, six (6/62, 10%) had cognition impairment or dementia with unspecified etiology (Table 3). All detected conditions are presented in Table 3.

**Table 3:** 62 patients with unlikely iNPH referred to KUH iNPH outpatient clinic from 2010 until 2017

	Number of patients (%)	% of all 253 study participants
Conditions	62 (100)	24.5
Alzheimer’s disease	14 (22.6)	5.5
Vascular dementia or cerebrovascular disease	11 (17.7)	4.3
Alzheimer’s disease and vascular dementia	7 (11.3)	2.8
Spinal stenosis	6 (9.7)	2.4
Cognition impairment or dementia with unspecified etiology	6 (9.7)	2.4
Ventriculomegaly (congenital or unclear etiology)	4 (6.5)	1.6
Drug induced Parkinsonism or psychiatric condition	4 (6.5)	1.6
Parkinson’s disease (1 idiopathic, 2 vascular)	3 (4.8)	1.2
Lewy’s bodies disease	2 (3.2)	0.8
Traumatic brain injury	2 (3.2)	0.8
Frontotemporal dementia	1 (1.6)	0.4
Vertigo	1 (1.6)	0.4
Arthrosis	1 (1.6)	0.4

*iNPH* Idiopathic normal pressure hydrocephalus, *KUH* Kuopio University Hospital

## Discussion

The Kuopio iNPH protocol is based on the two existing diagnostic guidelines in terms of the pre-treatment probability, classification and radiological analysis [1, 2], but there are some key differences in the prognostic tests used. While the protocol has emphasis on differential diagnostics, an ELD would have strengthened the prognostic value of the protocol. It is reasonable to assume, that some of the patients with negative LTT in this cohort might have benefitted from drainage test. There is a perplexing question: whether to downgrade the clinical probability for iNPH, as we have done, when competing diagnoses become more likely after the initial probability designation (Fig. 1). One could reasonably argue that the final clinical designation should be made before ancillary testing. Current diagnostic guidelines do not provide an answer to this question, but emphasize ruling out any other medical conditions at the start of classification, and to clinically follow those who exhibit negative prognostic/diagnostic tests [1, 2]. We argue that an option for re-classification, in addition to unified probability criteria, is needed. An additional difference from the established guidelines is that the cortical biopsy taken at surgery is part of the Kuopio iNPH protocol as a diagnostic and prognostic tool. The biopsy gives additional information for clinicians and helps patient and their families potentially to plan ahead if AD-related pathology is detected.

In clinical practice, the decision whether or not to perform shunt surgery is influenced not only by the results of the prognostic tests, but also by the clinical probability, representation and the accurate identification of iNPH. A variety of conditions share similar symptoms with iNPH [1, 2, 6] and may be seen at the outpatient clinic even if a preceding neurological evaluation has been performed (Table 3). This a priori patient selection has had an undoubtable effect on our results, since only a fourth of the patients had a condition other than iNPH. While these conditions were expected, it was interesting to see a heavily skewed distribution: in our cohort, other neurological conditions contributed to most of the differential diagnoses (74%, 46/62), the second largest group being musculoskeletal conditions (10%, 7/62) (Table 3). One could argue, that in terms of guideline classification, these patients should remain at least possible iNPH [1, 2]. We emphasize that while the competing condition was the most likely one in these patients, they can be referred for clinical re-evaluation if a suspicion of iNPH re-emerges.

In our experience, when a thorough differential diagnostics is performed, patients identified to have probable iNPH have a considerable possibility to benefit from CSF shunting even when their LTT and infusion test comes out negative [31]. In cases with more atypical presentation of iNPH, such as patients without gait impairment, infusion testing had a significant value as a differential diagnostic test. It is important to acknowledge, that delaying the treatment in patients with probable iNPH, due to e.g. long waiting times for surgery or unnecessarily extended diagnostic workup, can be harmful [7, 8]. In our cohort, iNPH patients presenting atypical symptomology significant comorbidities or other potential sources for their symptoms, underwent ancillary testing, the clear probable iNPH cases were directed to shunting right after the initial negative LTT. Nevertheless, the general outcome rate in our cohort was similar to that reported in the literature [32].

While a combination of the LTT and the infusion test perform well in identifying potential benefiters of shunt surgery, exclusion criteria based on these tests has not been presented [18]. A patient’s neurologic comorbidities, especially AD, may affect the gait response to LTT [31], and therefore the results of the LTT should be evaluated with care. Because the interpretation of the LTT may vary [2, 11–13], one could argue for a higher or a lower gait speed improvement threshold than we have used. While we have used a threshold based on our clinical experience, one could justifiably choose otherwise. Similarly, a longer observation time after LTT might be useful [7, 15, 17]. There exists an unsolved issue regarding the definition of a minimal clinically significant change in gait performance after the LTT. While out of scope to be fully addressed in this paper, we performed receiver operating characteristic (ROC) curve, placing the favorable 3-month iNPHGS –outcome as the binary variable [26]. Analysis did not show threshold for gait speed change (raw and percentage change) in LTT for this outcome indicator in this cohort with limited follow-up (Additional file 1: Figure S1). Further research in this area is needed.

While the gait performance is, by far, the most objective measure for outcome assessment in iNPH, other outcome indicators should accompany it. Performance in activities of daily living and patient reported outcome measures might enhance clinical evaluation in this regard [10, 33], but should not be used alone [33]. Unfortunately, we do not have follow-up information regarding the 14 probable or possible iNPH patients that were not shunted (Fig. 1). iNPH patients that are fit for surgery are encouraged to have shunt surgery after clinical re-evaluation, even after initial refusal.

## Conclusions

Our results emphasize the role of systematic diagnostic and prognostic workup especially in cases with an atypical presentation of iNPH e.g. without gait impairment as the leading symptom. Additional diagnostic testing may be required, but that should not delay adequate care. Active surgical treatment is recommended in patients with a high clinical probability of iNPH. Other neurological conditions contribute most of the differential diagnoses.

## Limitations and generalizability

The cut-off points between positive and negative prognostic tests as well as the performance of the tests vary between published studies. A number of limitations are identified in this study. A longer follow-up time would have strengthened the data. The LTT and the infusion test were performed from different lumbar punctures and ELD was not used in our protocol. Furthermore the neurosurgeon who reviewed the patient postoperatively was not, by rule, independent of the surgery.

## Additional file

**Additional file 1: Figure S1.** ROC analysis for 3-mo favorable iNPHGS outcome using gait speed change in LTT. Figure Legend: Favorable iNPHGS outcome is a reduction in the iNPHGS total score at least by a single point. Abbreviations: ROC, Receiver operating characteristic; AUC, Area under the curve; INPHGS, iNPH grading scale; iNPH, idiopathic normal pressure hydrocephalus.

## Data Availability

The anonymized datasets used and/or analyzed during the current study are available from the corresponding author upon reasonable request.

## Abbreviations

iNPH: idiopathic normal pressure hydrocephalus
CSF: cerebrospinal fluid
sNPH: secondary normal pressure hydrocephalus
NPH: normal pressure hydrocephalus
KUH: Kuopio University Hospital
CT: computed tomography
MRI: magnetic resonance imaging
LTT: lumbar tap test
TUG: timed up and go—test
ICP: intracranial pressure
iNPHGS: iNPH Grading Scale
MMSE: Mini-Mental State Examination
HPτ: hyperphosphorylated tau
Aβ: amyloid-beta
AD: Alzheimer’s disease
VAD: vascular dementia
PD: Parkinson’s disease
iPD: idiopathic PD
LBD: Lewy’s bodies disease
CVD: cerebrovascular disease
ml: milliliter
min: minute
mmHg: millimeter of mercury
ROC: receiver operating characteristic
