# Supplementary figures and images for: The Kuopio idiopathic normal pressure hydrocephalus protocol: initial outcome of 175 patients
Source: Fluids Barriers CNS. 2019 Jul 25;16:21. doi: 10.1186/s12987-019-0142-9 (PMC6657079; doi:10.1186/s12987-019-0142-9)

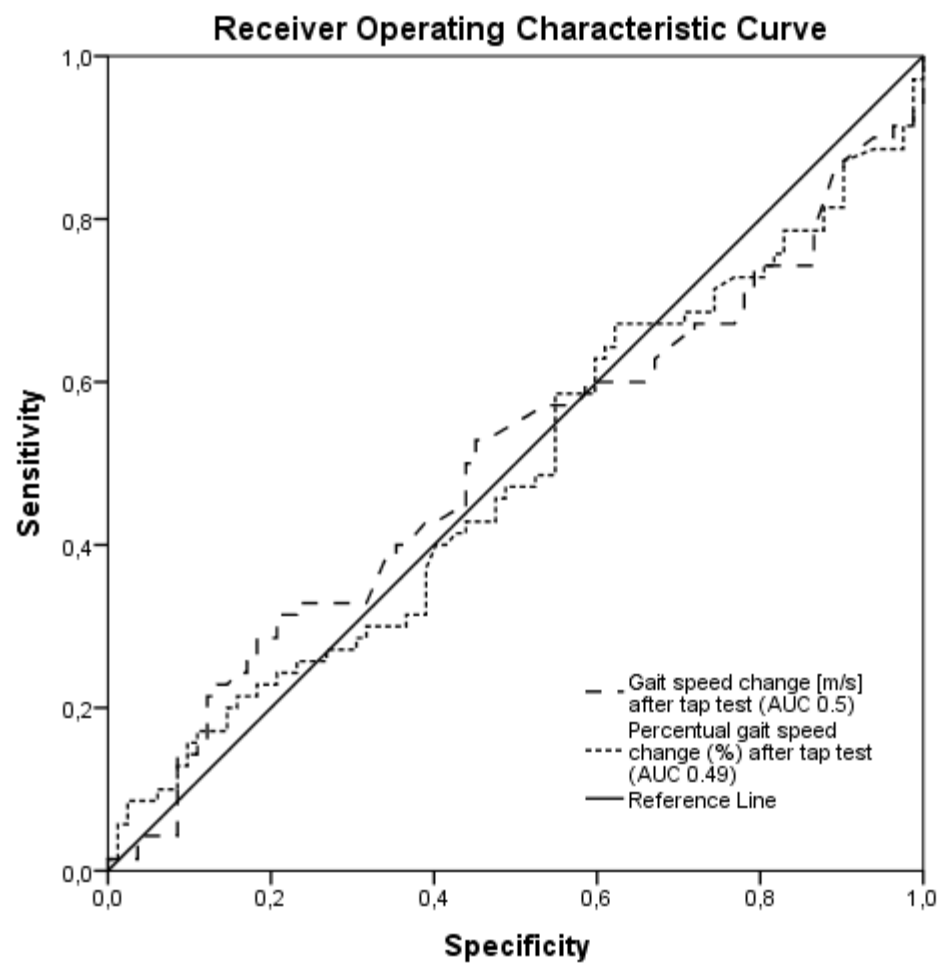

Supplement: Supplementary file 1 — Additional file 1: Figure S1. ROC analysis for 3-mo favorable iNPHGS outcome using gait speed change in LTT. Figure Legend: Favorable iNPHGS outcome is a reduction in the iNPHGS total score at least by a single point. Abbreviations: ROC, Receiver operating characteristic; AUC, Area under the curve; INPHGS, iNPH grading scale; iNPH, idiopathic normal pressure hydrocephalus. [file 12987_2019_142_MOESM1_ESM.pdf]
